# Interventions to improve ergonomics in the operating theatre: A systematic review of ergonomics training and intra-operative microbreaks

**DOI:** 10.1016/j.amsu.2020.02.008

**Published:** 2020-04-02

**Authors:** Kiron Koshy, Habib Syed, Andrew Luckiewicz, Daniel Alsoof, George Koshy, Lorraine Harry

**Affiliations:** a Royal Victoria Infirmary, Newcastle Upon Tyne Hospital Foundation Trust, Newcastle Upon Tyne, UK; b Brighton and Sussex Medical School, Brighton, East Sussex, UK; c University College London Medical School, London, UK; d Sancheti Institute of Orthopaedics and Rehabilitation, India; e Queen Victoria Hospital NHS Foundation Trust, East Grinstead, UK

**Keywords:** Ergonomics, Occupational injury, Surgical ergonomics, Operating theatre, Musculoskeletal injury

## Abstract

Musculoskeletal occupational injury is prevalent within the surgical community. This is a multi-factorial issue, but is contributed to by physical posture, environmental hazards and administrative deficiency. There is growing awareness of this issue, with several behavioural, educational and administrative techniques being employed. The literature on this topic is, however, sporadic and difficult to access by healthcare practitioners.

The aim of this systematic review was to evaluate the literature on the current interventions used to minimise musculoskeletal occupational injury in surgeons and interventionalists. This review will focus on administrative and human factor interventions, such as intra-operative microbreaks and ergonomics training.

## Highlights

• Musculoskeletal occupational injury is highly prevalent in surgeons and is well reported in the literature.
• No systematic reviews currently exist on interventions used to reduce musculoskeletal injuries in surgeons and interventionalists.
• In this study we carry out a systematic review to evaluate current interventions to minimise musculoskeletal in surgeons.
• This review is foccused on the use of ergonomics training and intra-operative microbreaks.

## 1 Introduction

Occupational or workplace injury has been recognised in the office environment for decades. However, this phenomenon is under-appreciated in the healthcare field. As an altruistic profession, healthcare workers often ignore their own physical health when providing care to patients. In addition to this, continually rising pressure to promote productivity, contributes to a culture that prioritizes output at the expense of healthcare professionals. The literature on musculoskeletal workplace injury is also not well appreciated in healthcare fields, likely due to a combination of sporadic research and lack of education.

Whilst a hazard in all disciplines of healthcare, some occupations are at particularly high risk [1]. It should come as no surprise that surgeons lie in this group, due in part to long periods of standing, bending and grasping in awkward positions. Other risk factors include equipment usage. For example, plastic surgeons often wear loupes which increase cervical loading by 40% [2], whilst other interventionalists regularly wear lead aprons which increase strain [3,4]: A 15-pound lead apron can put approximately 300 pounds per square inch of initial pressure on the intervertebral disc strain: [[5], [6], [7], [8]].

Specific procedures also appear to carry a greater risk of musculoskeletal injury. Minimally invasive surgery (MIS) in particular has been reported as carrying a greater risk of musculoskeletal injury [5,[9], [10], [11], [12], [13], [14], [15], [16], [17], [18], [19], [20], [21], [22], [23], [24], [25], [26]], when compared to conventional open surgery. This is likely due to long periods of static posture in ergonomically poor positions.

In the last decade, this issue has been increasingly recognised. A recent meta-analysis found that 68% of surgeons reported generalised pain [27] and a study by Park et al. found that up to 87% of surgeons performing MIS experienced work-related pain [28]. Diagnoses of disc prolapse have also been found to be as high as 15% in study populations [29]. The most common anatomic sites affected were the back (50%), neck (48%) and arm or shoulder (43%). Fatigue, stiffness and numbness were also prevalent symptoms.

The quality of life of a surgeon is also affected by their musculoskeletal discomfort. One study found that 41% of their participants felt their pain interfered with their relationships, and 51% reported a disturbance in their sleep which can decrease cognitive capacity and subsequently lead to increased surgical errors [13]. These disturbances combined with a poor work life balance may culminate in surgeon burnout.

Operating exacerbated pain in 61% of surgeons, but only 29% sought treatment for their symptoms [27]. However, individual studies have reported as many as 31% of arthroplastic surgeons required surgery for their musculoskeletal injuries [30].

Importantly, one survey demonstrated that of surgeons with past musculoskeletal complaints, 26.7% required work leave and 40.0% made intraoperative adjustments [31]. This issue has, therefore, significant financial and workforce implications.

There are also psychological ramifications. A survey found that 47% of surgeons were concerned that these conditions will shorten their career [32]. This fear is not unfounded, however, as a survey of ophthalmic plastic surgeons reported that 9.2% stopping operating due to pain or spinal injury [33].

Poor health in surgeons, undoubtedly affects patient care. 30% of surgeons said that they took their own physical symptoms into account when recommending a surgical approach for their patients [27]. Furthermore, a recent survey found that students are less likely to enter surgical careers due to musculoskeletal ergonomics issues [34].

To tackle this issue, various ergonomic solutions have been proposed. These can be split into three categories: engineering controls; administrative controls and personal protective equipment.

Engineering controls are changes that can be made in the physical theatre environment; this includes structural changes such as bed height and equipment changes, such as the use of floor mats.

Administrative controls are workforce or human changes. These include taking breaks during operations and ergonomics training.

Personal protective equipment are tools individual staff may use, such as lighter lead aprons or body support equipment.

These three categories of control intertwine and each plays a role in potential improvement. In this study, we aim to perform a systematic review of the literature on administrative interventions used to reduce musculoskeletal occupational injury in surgeons. The focus has been applied to the use of intra-operative microbreaks and ergonomics training. This is because these interventions are internationally available and require a relatively small amount of resources to incorporate into practice if found to be beneficial.

## 2 Methods

### 2.1 Systematic review

The review was performed in accordance to the PRISMA statement. The study protocol was established and published prior to conducting the review.

### 2.2 Information sources and search

The literature search was carried out between September 2017–July 2019. The literature search was performed using EMBASE, MEDLINE, CINAHL, Google scholar, Cochrane library and NICE database. Relevant natural language and controlled vocabulary terms were selected and combined. Final result sets were de-duplicated and reviewed for relevance by the searcher, irrelevant results being discarded. The articles were then screened by title and abstract.

Natural language terms used included, for occupational injuries the strings, ((occupational OR workplace*) ADJ2 (injur* OR symptom)), ((musculoskeletal OR muscle* OR back OR neck) ADJ2 (injur* OR symptom* OR pain OR ache* OR fatigue* OR tired*)), backache, and for ergonomic interventions the strings (ergonomic* ADJ2 (intervention* OR modification*)), (“floor mat*"), (“body support*"), (ergonomic* ADJ2 (chair* OR seat*)), (theatre* ADJ2 design), (“operating room*" ADJ2 design), (“lead apron*"), and (microbreak* OR “micro-break*" OR micropause* OR micro-pause*).

### 2.3 Inclusion criteria

Studies investigating peri-operative ergonomic interventions in the operating theatre
Studies utilising administrative interventions to reduce musculoskeletal occupational injury
Studies that have implemented an administrative intervention as part of the study design

### 2.4 Exclusion criteria

Studies reporting on operative work that took place outside of a hospital operating theatre
Studies reporting on non-medical staff
Studies reporting on the use of bespoke or specialised equipment
Studies that have not implemented ergonomics training or a form of administrative intervention

### 2.5 Intervention(s)/exposure(s)

Administrative controls are workforce or human changes. These include taking breaks during operations and ergonomics training. Five of these described the use of intraoperative microbreaks as an ergonomic intervention. Two studies investigated the use of ergonomics training.

## 3 Results

**Fig. 1:**
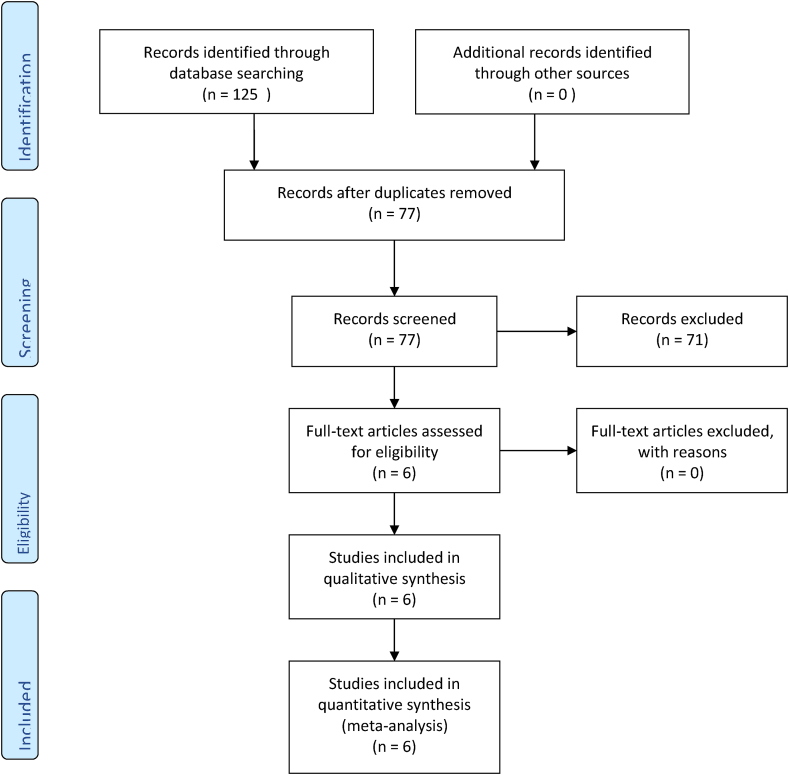
PRISMA flow diagram for the selection of studies.

Six full text articles fulfilled the inclusion and exclusion criteria and were incorporated into the present study [13,15,[35], [36], [37], [38]] (Fig. 1). Of these, four studies investigated the use of intra-operative microbreaks [13,15,35,37]. Two studies investigated the use of ergonomics training [36,38]. Table 1 contains a detailed description of the studies.

**Table 1:** Detailed overview of the studies included in the review.

First Author	Title of Abstract	Type of study	Number of participants	Study population	Intervention	Results
**Hallbeck et al.** [13]	The impact of intraoperative microbreaks with exercises on surgeons: A multi-centre cohort study	Non-randomised cross over study	56	Surgeons in the following specialties: General, Paediatric, Orthopaedic, neurosurgery, Urology, Otorhinolaryngology, Gynaecology, Plastics, Thoracic and Vascular surgery	60–90 s guided Intraoperative microbreaks with exercises performed within the sterile field at medically convenient 20–40 min intervals.	(1) Statistically significant improvement in shoulder discomfort with incorporation of microbreaks. (2) Mental focus improved in 34.4%, remained the same in 53.3% and diminished in 12.4%. (3) Physical performance improved in 57.4%, remained the same in 42.6% and diminished in 0%. (4) Perceived impact of microbreaks was minimal with participants giving a median rating of 2/10. (5) 87% would want to incorporate microbreaks into their surgical routine (6) The intervention did not prolong the operative length.
**Komorowski et al.** [15]	The influence of micropauses on surgeons' precision after short laparoscopy procedures	Randomised controlled trial	2	General surgeons	30 s intraoperative microbreaks every 15 min. Turn away from workstation and patient, stretch neck, shoulders and hands	(1) Average number successful trials greater for appendectomy procedures compared to cholecystectomy. (2) No statistical difference in the precision between the intervention and control group.
**Dorion and Darveau.** [35]	Do Micropauses Prevent Surgeon's Fatigue and Loss of Accuracy Associated with Prolonged Surgery? An Experimental Prospective Study	Non-randomised crossover study	16	Surgeons in the following specialities: General, Neurosurgery, Head and Neck and Cardiac	20 s intraoperative microbreaks every 20 min, using alarms. Stretch the neck and shoulders.	(1) Significantly less discomfort in all body areas in the micropauses group compared to the non-micropauses group. Only non-significant areas were eyes and legs. (2) Statistically significant improved function (strength) with micropauses. (3) Statistically significant improvement in surgical accuracy in the microbreak group.
**Engelmann et al.** [37]	Effects of intraoperative breaks on mental and somatic operator fatigue: a randomised clinical trial.	Randomised controlled trial	7	Paediatric surgeons	25 min work period followed by 5 min of unstructured breaks	(1) Cortisol levels 22% higher in surgeons without breaks. P < 0.05. (2) Event related cortisol higher in the control group P < 0.05. (3) Significantly more intraoperative events in control group. (4) Participants in break group performed significantly better in tests assessing concentration and performance. (5) Significantly decreased levels of fatigue from pre- to post-op in break group. Reported decrease in perceived stress. Musculoskeletal strain and pain scores: significant improvements p < 0.001 in upper extremities locomotive and trunk's static elements. Non-sig for eye strain.
**Reddy et al.** [36]	The impact of the alexander technique on improving posture and surgical ergonomics during minimally invasive surgery: pilot study	Prospective cohort study	7	Urology surgeons	Alexander technique	(1) Participants reported improvement in posture and discomfort. (2) Statistically significant improvement in postural assessment of 5 postAT postural measurements vs preAT values, including the time load test (p = 0.04). (3) Nondominant hand showed statistically significant improved intentional tremor score. (4) Decreased perceived discomfort and fatigue at baseline and during the FLS modules postAT, - not statistically significant. (5) Majority of subjects had improvement in postAT FLS scores in 3/4 modules with 2 values being statistically significant. (6) Reported decrease in perceived effort in performing modules with 2 values showing statistical significance.
**Franasiak et al.** [38]	Feasibility and effectiveness of an ergonomics training program to address high rates of strain among robotic surgeons	Non-randomised cross over study	42	Robotic surgeons: Urology, Obstetrics & Gynaecology and Otorhinolaryngology	In person/online ergonometric training designed by a expert human factor engineer using occupational safety and health guidelines.	(1) 88% of participants changed practice following the ergonomics training (ET) programme. (2) 84% of participants with previous strain reported reduction in strain post ET.

Study types included randomised controlled trials [15,37]; crossover studies [13,35] and cohort studies [36,38].

For microbreaks, number of participants included ranged from two [15] to 56 that completed the study [13]. Ergonomics training participants ranged from seven [36] to 38 that completed follow up [38].

### 3.1 Participants

Surgical speciality representation was varied and included: paediatric surgeons (37), urological surgical trainees [36] and a mixture from general, paediatric, orthopaedic, neurosurgery, urology, otorhinolaryngology (ENT), gynecology, plastics, thoracic, vascular surgery, neurosurgery, cardiac, and robotic surgeons [13,35,38].

### 3.2 Intra-operative microbreaks

#### 3.2.1 Definition

Various definitions were used in the extracted studies of the term ‘microbreaks’. Hallbeck et al. described a 1.5–2 min break with guided microbreak exercises carried out intraoperatively in the sterile field at medically convenient 20–40 min intervals. Exercises focused on neck, back, shoulders, hands and lower extremities [13,32].

Komorowski et al. have incorporated a 30 s break every 15 min, where surgeons turn from work station and the patient, to stretch the neck, shoulders and hands [15]. Dorion et al. also described a highly structured microbreak with alarms every 20 min to signify a 20 s break to stretch the neck and shoulders [35]. Engelmann et al. used a less structured approach with 25 min work followed by 5 min unstructured breaks [37].

#### 3.2.2 Outcome measures and results

Due to the heterogeneity of methods, the outcome measures and results will be described individually.

Hallbeck et al. assessed musculoskeletal pain using ‘body part discomfort’ scales [13]. This study found significantly improved self-reported pain in the shoulders when using intra-operative microbreaks. 57% of participants reported an improvement in physical performance and 38% reported an improvement in mental performance. 87% of participants wanted to utilise microbreaks into their future practice. Interestingly, despite the fact that 73.5% of participants reported a non-zero effect of distraction due to the exercise and 78.9% reported a non-zero effect on flow of the operation from the exercises, this study found no significant difference in operation time when microbreaks were utilised.

Komorowski et al. assessed precision and accuracy using a mobile application called ‘reverse maze’ [15]. They assessed the number of successful trials and mean time of successful trials. No significant difference was found between the experimental group utilising microbreaks and the control group.

Dorion and Darveau assessed muscular fatigue by using a visual analogue scale and measuring the time a 2.5 kg weight could be held [35]. Accuracy was assessed by measuring the number of errors in cutting a a star patter with Metzenbaum scissors. Results found a significantly reduced subjectively reported body discomfort in all body areas, with non-significant findings in the eyes and legs. Accuracy was also significantly improved in the group taking microbreaks.

Engelmann et al. investigated biochemical markers and found significantly lower levels of cortisol in participants using microbreaks [37]. There were also fewer intra-operative events and peaks of α amylase in the microbreaks group. In addition to this, error rates in the BP concentration test were fourfold lower in the microbreaks group. There was also no significant difference in operative time.

#### 3.2.3 Ergonomics training

Reddy et al. investigated the ‘Alexander technique’ (AT) of ergononomics training [36]. They assessed self-reported musculoskeletal pain scores which found no significant difference pre and post-AT. Posture was assessed with an AMSAT score and found no significant difference pre and post-AT. Objective biometric data measured included blood pressure, heart rate, height, foot length, and wingspan which showed no significant difference pre and post-AT. There was however, a significantly reduced resting respiratory rate post and increased peak inspiratory chest circumference post-AT.

Secondary outcome measures assessed performance on ‘Fundamentals of laparoscopic surgery’ (FLS) laparoscopic modules. The study found various measures to be significantly improved as per Table 2, however, the majority of outcomes showed non-significant improvement.

**Table 2:** PreAT and postAT intentional tremor and manual dexterity, perceived and baseline discomfort, and FLS and perceived effort scores.

	**Mean PreAT Score**	**Mean PostAT Score**	**P Value**
***Hand tremor + dexterity***			
**Tremor:**			
**Rt**	16.3571	14.098	0.1111
**Lt**	19.5586	15.1343	0.0269^a^
**Dominant**	16.2	14.101	0.1189
**Nondominant**	19.75	15.12	0.023^a^
**Dexterity (secs):**			
**Rt**	17.71	17.11	0.2876
**Lt**	18.93	18.20	0.2564
***Discomfort***			
**Baseline pain:**			
**Neck**	2.57	1.14	0.2287
**Back**	2.57	1.14	0.2287
**Perceived pain during FLS modules:**			
**Neck**	2.80	3.00	0.3739
**Shoulder**	3.00	1.60	0.3508
**Upper back**	1.00	0.60	0.4766
**Perceived fatigue during FLS modules**	1.00	0.50	0.178
***FLS + effort***			
**Time to complete (mins):**			
**Bead transfer**	2.97	2.45	0.4788
**Cutting circle**	7.88	5.97	0.0891
**Placing suture**	6.14	4.41	0.0891
**No. Beads dropped**	4.71	1.71	0.0459^a^
**No. Rings:**			
**Transferred**	5	9	0.0314^a^
**Dropped**	1	1.86	0.2695
**Module perceived effort:**			
**Bead transfer**	60.29	41.71	0.1730
**Ring transfer**	72.67	30.33	0.0429^a^
**Circle cutting**	101	86	0.1885
**Suturing**	103	64.33	0.0071^a^
***Hand tremor + dexterity***			
**Tremor:**			
**Rt**	16.3571	14.098	0.1111
**Lt**	19.5586	15.1343	0.0269^a^
**Dominant**	16.2	14.101	0.1189
**Nondominant**	19.75	15.12	0.023^a^
**Dexterity (secs):**			
**Rt**	17.71	17.11	0.2876
**Lt**	18.93	18.20	0.2564
***Discomfort***			
**Baseline pain:**			
**Neck**	2.57	1.14	0.2287
**Back**	2.57	1.14	0.2287
**Perceived pain during FLS modules:**			
**Neck**	2.80	3.00	0.3739
**Shoulder**	3.00	1.60	0.3508
**Upper back**	1.00	0.50	0.178
**Perceived fatigue during FLS modules**	1.00	0.50	0.178
***FLS + effort***			
**Time to complete (mins):**			
**Bead transfer**	2.97	2.45	0.4788
**Cutting circle**	7.88	5.97	0.0891
**Placing suture**	6.14	4.41	0.1141
**No. Beads dropped**	4.71	1.71	0.0459^a^
**No. Rings:**			
**Transferred**	5	9	0.0314^a^
**Dropped**	1	1.86	0.2695
**Module perceived effort:**			
**Bead transfer**	60.29	41.71	0.1730
**Ring transfer**	72.67	30.33	0.0429^a^
**Circle cutting**	101	86	0.1885
**Suturing**	103	64.33	0.0071^a^

a Statistically significant.

Franasiak et al. used a bespoke ergonomics training programme (ET) for robotic surgeons [38]. They assessed self-reported change in practice and found that 88% of participants changed their practice as a result of the ET. Key areas include change in chair height to allow 90° of knee flexion (75% of this group); adjustment of armrest to allow forearm position to be parallel to the floor (67.9%) and adjusting head tilt to allow no more than 20° of neck flexion (50%). This study also assessed self-reported muscular strain and found 74% of participants that reported pain prior to the intervention, noticed a reduction following the ET.

## 4 Discussion

Ergonomics is a body of knowledge about human abilities, human limitations and human characteristics that are relevant to design. Ergonomic design is the application of this body of knowledge to the design of tools, machines, systems, tasks, jobs and environments for safe, comfortable and effective use. Whilst utilised extensively in the corporate sector, less ergonomics work has been carried out in the health care field. This systematic review has focussed on investigating the effect of administrative ergonomics, namely intra-operative microbreaks and surgical ergonomics training. This is because these interventions are internationally available and require a relatively small amount of resources to incorporate into practice.

### 4.1 Microbreaks

Microbreaks are a widely used strategy to mitigate occupational risk of musculoskeletal injury and to enhance workplace performance, especially in sedentary occupations. However, the reported use of them within the surgical field is limited. In our review, four studies met our inclusion/exclusion criteria. All studies demonstrated microbreaks to be beneficial to surgeons through multiple domains, from reduced reported muscle discomfort to improved mental focus and surgeon overall well-being.

The need for a surgeon to be accurate and precise during a surgical procedure is paramount in order to minimise risk of harm to patients. Past studies have shown longer surgical procedures cause greater muscular fatigue and therefore, impairs a surgeon's technical accuracy and precision. This in particular was highlighted by Dorion and Darveau who found participants in the non-microbreak group had on average a seven-fold increase in error rate when performing a star-shaped precision test compared to the microbreaks group [35]. However, the validity of this test is unknown and how it translates into real practice is yet to be determined. The study by Komorowski et al. provided contrasting results when relating microbreaks to surgical precision [15]. They found no significant difference in speed or precision of the surgeon when incorporating microbreaks into their surgical routine. However, it is worth noting that this study is significantly limited by its sample size of two and did not investigate the effect on surgeon pain. Also, precision was measured using a simple mobile phone application which was not clinically validated. This study was included in the present review because of its interesting use of precision measures, which could be easily repeatable. However, the findings should be interpreted with caution due to these mentioned limitations.

The cause of the decrement in the surgeon's performance extends beyond muscular fatigue. Evidence has shown surgeons are at higher risk of musculoskeletal injury, particularly MIS surgeons, as their role involves adopting unorthodox positions and postures for long periods of time [41]. The significance of this can be appreciated as a study of 260 surgeons found that 53% of their injured surgeons reported that the pain from their injury had a mild to moderate effect on their surgical performance, therefore, potentially affecting the quality of surgical care [42]. However, this has not been formally assessed by previous studies and therefore it would be useful for future studies to address. Overall, the studies have demonstrated that microbreaks are an effective method of reducing muscular fatigue and mitigating occupational risk of injury.

The are some perceived challenges to introducing microbreaks. There is the question as to whether the benefits of microbreaks to the surgeons compromise the quality of care provided to the patients. Workflow interruptions are a potential issue cited by critics of microbreaks. It is believed that these breaks create distractions which could possibly lead to increased surgical errors as demonstrated by previous studies focusing on the effect of non-routine events on surgical performance [43]. However, individual studies conducted by Hallbeck et al. and Park et al. demonstrated that microbreaks had a minimal/no impact; surgeons in Hallbeck's study gave a median rating of impact as 2/10 [13]. In fact, microbreaks may be beneficial to operative flow. Zheng et al. reported that on average, operative flow is interrupted 4.1 min per hour due to trivial tasks such as equipment/shift change, and personal tasks such as responding to messages [44]. Therefore, as observed in both Hallbeck's and Park's study, microbreaks allow for these non-routine events to be addressed minimising their impact on operative flow.

Microbreaks have also been shown to have a positive impact on communication within a team. One study found it enabled more detailed explicit instructions to be relayed to members of the surgical team, also giving time for detailed feedback. This is particularly important when performing complex procedures as implicit communication in these non-routine surgeries can result in fatal errors [45]. Furthermore, studies have shown that primary tasks with a high workload, for example, dissection, reduce the capacity for communication within a team. Microbreaks decrease primary task workload allowing more time for communication between team members minimising the risk of fatal errors [40].

Establishing microbreaks as part of a surgical routine may be challenging due to the attitudes and beliefs held by some surgeons. This was evident by the low compliance in taking regular microbreaks by some surgeons in the studies. A survey of surgeons in Engelman's study found the majority of the surgeons felt neutral or negative about their proposed microbreak protocol despite the proven benefits of the protocol to a surgeon's performance and overall health [40]. Dorion and Darveau suggested that the low compliance may be due to surgeons “feeling of invincibility, lack of awareness, touch of laziness and a leave-me-alone attitude” [35]. Being able to complete a number of operations within a defined amount of time is a source of pride within the surgical culture. Therefore, microbreaks may be perceived by some surgeons as a trivial intervention which prolongs the length of the surgery and decreases the number of operations, they can perform in a certain time interval. Observations by Engelman et al. supports this theory as they found surgeons who perceived themselves to be fast (≥5 operations), rated the breaks significantly lower than slow operators [40]. Nonetheless, studies conducted by Engelman and Park et al. have discredited this belief as they demonstrated microbreaks to be beneficial to surgeons and patients without prolonging the overall operating time [37]. To overcome this obstacle, as suggested by Dorion and Darveau, the nursing staff should take responsibility in enforcing the breaks – this was proven to be an effective measure in Komorowski study [15,35].

The optimal duration and timing of the breaks is difficult to discern, as it differed between the studies. Dorion and Darveau chose 20 s breaks every 20 min [35], as a study from Rohmert found the time taken to recuperate during a rest is exponentially related to the level of fatigue and recuperation is quicker at earlier intervals of the break. The surgeons in their study felt the breaks were initially too frequent, however, after 1 h, they were welcomed. It is possible that 1-min breaks every 20–40 min, as proposed by Hallbeck, would be an improvement, as this would allow sufficient time for all members of the team to perform adequate stretches, whilst a 20 s interval may be insufficient for some. This is supported by the results of a self-reported survey from MIS surgeons in previous studies. They found that breaks with 1-min duration and ones that could be performed without breaching the sterile field were preferred. This minimises the impact on operative flow and ensures the surgical team remains close to the operating theatre as in previous studies participants expressed difficulty in getting everyone “back on the deck after the break [40]. Although, it is important to note, Engelman et al. had success with 5-min breaks every 25 min, the participating surgeons’ views on the length and regularity of the breaks was not reported.

The structure of the breaks also varied between the studies. Engelman utilised unstructured breaks without any stretching exercises. However, the benefits of simple rest breaks without exercises are limited and may be inadequate in relieving the stress on the joints [46,47]. The remaining studies used either stretches or guided exercises focusing on specific parts of the body, particularly the neck, back and shoulders as these have been shown to be the most common sites for musculoskeletal symptoms [27]. Evidence suggests dynamic active stretching exercises are more effective than static stretches [48]. It is also important to ensure the stretches are easy to follow and can be performed adequately within the allocated time. Good examples of dynamic exercises have been described in previous studies [32,46].

### 4.2 Ergonomics training

There have been very few studies investigating the effect of ergonomics training in surgery, with only two meeting the inclusion/exclusion criteria. The study investigating the Alexander technique, which is a validated ergonomics programme found no overall significant difference in the majority of its outcomes. This study was, however, likely to be underpowered, with only seven participants.

The study by Franasiak et al. however, found that after using a bespoke surgical ergonomics programme, there was a significantly improved self-reported level of pain and the majority of participants would implement the programme into their practice. This study also benefitted from a larger sample of 42 participants, however, the study design makes direct comparison difficult. Bespoke ergonomics programmes provide a more tailor-made exercise regime based on the participants’ current understanding, operating equipment and physical debilitations. In this sense, they are likely to provide improved outcomes and match more closely the advice provided by specialist allied professionals. However, this approach does lack the test-retest reliability of validated methods like the alexander technique. This is a general issue in this field, which is limited by the lack of standardisation in ergonomics training, both in terms of content and delivery. Further work needs to be carried out to compare methods of training and allow improved standardisation or ergonomics training regimes.

There does appear to be a growing wish for ergonomics training in the surgical field. A study found that only approximately one-third of survey respondents (33.2%, n 5125) acknowledged that they learned ergonomic principles in training [39]. Surgeons, who performed endoscopic surgery and pursued treatment for pain, were significantly less likely to have been taught ergonomic principles during their training. Furthermore, 21% independently sought out information regarding ergonomic principles and specific recommendations for surgeons. Among those who implemented these principles in their surgical practice, the majority (69.6%) noted improvement in their musculoskeletal symptoms.

There is increasing research into developing ergonomic interventions to reduce risk factors for musculoskeletal disorders associated with surgery. Despite this, the use of ergonomic interventions in practice is still limited. This in part, may be due to surgeons often working under strict time constraints, meaning they do not have time to consider ergonomic alterations while operating. Also, the strict regulation for the design of surgical equipment limits the scope for the optimal ergonomic design [40]. Limited education and awareness of ergonomic interventions may also be a reason for the poor compliance as a recent meta-analysis reported 59%–99% of surgeons failed to recall their institutions ergonomic recommendations and none had received any particular ergonomic training [27].

## 5 Limitations

No systematic assessment of bias has been carried out.

Number of participants (n), has been low in all the studies included. This may limit the external validity of these results and suggest that larger studies are required. No meta-analysis has been carried out, due to the heterogeneity of data in studies included.

## 6 Conclusion

Occupational injury in healthcare is a long-neglected, multifactorial and very prevalent issue, with reported 68% of surgeons suffering with generalised pain [27]. In this review we set out to explore administrative approaches to reduce MSK injuries in surgeons. We found that ergonomic training can be a very accessible and effective way of achieving that goal, with up to 69.9% of surgeons noting improvement in their symptoms. There is a consistent body of evidence to suggest that microbreaks are an effective ergonomic intervention with proven benefits to surgeons and patients. Standardisation, large-scale studies and validated assessment methods are however still lacking, suggesting that further work is required to validate these interventions and ensure effectiveness as they are introduced on a widespread basis.

## Ethical approval

Ethical approval not required.

## Sources of funding

No funding required.

## Author contribution

Koshy K – Study design, data collection, data analysis, writing.

Syed H – Data collection, data analysis, writing.

Luckiewicz A – Data collection, data analysis, writing.

Alsoof D – data analysis, writing.

Koshy G – Data analysis, writing.

Harry L – Study design, writing.

## Trial registry number

Name of the registry: Research Registry.

Unique Identifying number or registration ID: reviewregistry805.

Hyperlink to the registration (must be publicly accessible): https://www.researchregistry.com/browse-the-registry#registryofsystematicreviewsmeta-analyses/?view_13_search=alsoof&view_13_page=1.

## Guarantor

Mr Kiron Koshy.

## Disclaimer

The authors, their immediate families, and any research foundation with which they are affiliated did not receive any financial payments or other benefits from any commercial entity related to the subject of this article.

## Provenance and peer review

Not commissioned externally peer reviewed.

## Declaration of competing interest

None to report.
